# TGV-regularized inversion of the Radon transform for photoacoustic tomography

**DOI:** 10.1364/BOE.379941

**Published:** 2020-01-22

**Authors:** Kristian Bredies, Robert Nuster, Raphael Watschinger

**Affiliations:** 1 Institute of Mathematics and Scientific Computing, University of Graz, Heinrichstrasse 36, 8010 Graz, Austria; 2 NAWI Graz and BioTechMed Graz, Mozartgasse 12/II, 8010 Graz, Austria; 3 Department of Physics, University of Graz, Universitaetsplatz 5, 8010 Graz, Austria; 4 Institute of Applied Mathematics, Graz University of Technology, Steyrergasse 30, 8010 Graz, Austria; 5 kristian.bredies@uni-graz.at; 6 ro.nuster@uni-graz.at; 7 watschinger@math.tugraz.at

## Abstract

We propose and study a reconstruction method for photoacoustic tomography (PAT) based on total generalized variation (TGV) regularization for the inversion of the slice-wise 2D-Radon transform in 3D. The latter problem occurs for recently-developed PAT imaging techniques with parallelized integrating ultrasound detection where projection data from various directions is sequentially acquired. As the imaging speed is presently limited to 20 seconds per 3D image, the reconstruction of temporally-resolved 3D sequences of, e.g., one heartbeat or breathing cycle, is very challenging and currently, the presence of motion artifacts in the reconstructions obstructs the applicability for biomedical research. In order to push these techniques forward towards real time, it thus becomes necessary to reconstruct from less measured data such as few-projection data and consequently, to employ sophisticated reconstruction methods in order to avoid typical artifacts. The proposed TGV-regularized Radon inversion is a variational method that is shown to be capable of such artifact-free inversion. It is validated by numerical simulations, compared to filtered back projection (FBP), and performance-tested on real data from phantom as well as in-vivo mouse experiments. The results indicate that a speed-up factor of four is possible without compromising reconstruction quality.

## 1. Introduction

Photoacoustic (or optoacoustic) tomography (PAT) is a promising imaging technology that combines the favorable properties of pure optical and ultrasound imaging methods. In general, PAT images reveal a contrast determined by the absorption of pulsed laser light by natural endogenous chromophores (e.g. hemoglobin, lipids) or by injected contrast agents (e.g. chromophores, gold nano-particles) in biological samples. The conversion of the absorbed energy into ultrasound occurs via the thermoelastic effect. Thereby, excited and expanding ultrasound waves are monitored outside the sample and the contained information is used for image reconstruction. Compared to other imaging modalities, PAT is a non-ionizing imaging modality with superior blood-vessel contrast suitable for frequent screening purposes and provides higher spatial resolution up to larger imaging depths compared to pure optical imaging. The asset and operating costs are low compared to MRI systems. Examples of application areas of PAT are versatile such as cancer diagnoses, neuronal imaging, hemodynamics monitoring and atherosclerotic plaques detection [1–4].

Current PAT systems differ in terms of ultrasound detection principle (e.g. piezoelectric, optical), detection geometry (planar, spherical, linear, circular or arc), shape and size of used detectors with the accompanied pros and cons of each implementation related to the delivery of the excitation light, limited view problem, detection sensitivity [2,4–7]. While most of the tomographic systems are implemented with ultrasound detection elements with dimensions smaller than the object size, Burgholzer et. al [8] introduced the concept of using integrating area and line detectors in PAT. This has the advantage that the signals are exact projections over areas or lines enabling the use of numerically efficient reconstruction algorithms, such as the inverse Radon transformation. In practice, the integrating line detector concept is preferable over large area detection due to the easier technological implementation and the parallel detection capability. PAT systems were built with 64 piezoelectric and optical fibers arranged as one dimensional (1D) array along a half circle to record integrated time resolved pressure signals from various directions simultaneously [9,10]. Thereby, projection images from the initial pressure source are gathered in almost real-time only limited by the need of multiplexing due to the limitations of data acquisition (DAQ) channels. As an alternative that does not require external electronics such as amplifiers and analog-to-digital converters for each channel, we investigated the use of a charge-coupled device (CCD) camera combined with an optical phase contrast technique for acoustic detection [11,12]. The camera records projection images of the diverging wave pattern at a defined wave propagation time. Hence, the information in recorded snapshots of the acoustic field is purely spatially instead of temporally.

In general, the procedure to form a 3D image from projection data is separable into two steps. First, the recording of integrated temporal pressure signals or acoustic wave pattern images from several directions perpendicular to the rotation axis, where the data of each orientation is used to reconstruct a 2D initial pressure distribution. The second step is to apply the inverse Radon transform to the calculated projection data set such as in X-ray computed tomography. For the reconstruction of the 2D initial pressure distributions from temporal pressure signals in the first step, various direct methods are available, such as back projection [13,14], time reversal [15,16], or frequency-domain [13,17,18] algorithms. Although each of these methods can be adapted to spatial data, back propagation in frequency space [11,19,20] is often applied due to its numerical efficiency and simple implementation.

The 3D imaging speed of currently implemented imaging systems is in the range of 20 seconds and mainly limited by the sequential recording of the projection data from various directions of the sample. Aiming at a certain resolution and imaging quality within a defined area, the amount of projection data (orientations) is defined by the Nyquist sampling criterion when using direct reconstruction methods such as the inverse Radon transform. A reduction of imaging time towards real-time 3D PAT by parallelization, i.e., the simultaneous recording of several projection directions, is very challenging with integrating detection schemes due to the lack of space surrounding the sample.

Nevertheless, to investigate fast dynamic processes, we study to which degree it is possible to speed up 3D imaging time by using less measured data and employing variational methods for Radon transform inversion. The reasons for choosing variational regularization in this context are threefold. First, approaches based on Tikhonov functional minimization are well-studied in terms of stability with respect to noisy measurements and convergence for vanishing noise [21]. Second, with respect to Radon inversion, the use of sophisticated image reconstruction methods allows a reduction of the amount of projection directions without introducing image distortion and stripe artifacts [22]. And third, appropriate regularization functionals have already been successfully employed for few-view computed tomography, for example, the *total variation* (TV) [23,24]. Nevertheless, other regularization strategies exist, such as Bayesian inversion or inversion based on deep learning [25]. In particular, the latter has recently been analyzed with respect to regularization of ill-posed inverse problems [26].

In the context of variational regularization, TV is known for its edge-preserving properties [27] and good regularization performance for inverse problems in imaging [28]. Total-variation-based variational approaches have already successfully been employed for photoacoustic tomography [29–32], but, to the best knowledge of the authors, only for point detectors and not for line-integral detection strategies such as integrating line detectors or optical phase contrast techniques. While TV-based approaches are generally useful for tomographic reconstruction from incomplete data, one of their limitations is the tendency of TV to prefer piecewise constant images, which is often not realistic and leads to artifacts, the so-called *staircase effect*. Many strategies for the reduction of these artifacts have been proposed (such as, e.g., [33], but also regularizers involving wavelet/curvelet transforms [34] or patch-based methods [35]), among which the *total generalized variation* (TGV) constitutes an effective regularizer [36] which is convex, edge-preserving and well-suited for inverse imaging problems [37,38]. In particular, TGV is an established and adequate model for medical images with many successful applications being reported in the medical imaging literature [39–45]. Also, recently, the potential of TGV-regularized photoacoustic tomography with point detectors has been demonstrated [46], and CT/Radon inversion with total generalized variation regularization has been established in the context of X-ray [47] and electron tomography [48].

In this paper, we show the benefits of TGV-regularized Radon inversion applied to photoacoustic tomography with line-integration strategies, in particular, for optical phase-contrast PAT imaging. To the best knowledge of the authors, this is the first time that in this context, a full 3D variational reconstruction is performed and validated, both numerically and experimentally, and using a sophisticated image model, a realistic data set size as well as a sufficiently high image resolution. In particular, regarding few-angle measurements, the proposed approach has the potential to push the limits for optical phase-contrast PAT.

The article is organized as follows. In Section 2, we briefly introduce the mathematical model which describes the photoacoustic imaging process in [11,12]. We present a solution strategy which leads to the problem of inverting the Radon transform. For the latter, we propose TGV-regularized inversion which yields a non-smooth, convex minimization problem. In Subsection 2.1, we discuss how such problems can be solved in general, while a concrete algorithm is given in Subsection 2.2. In Section 3, a computational validation of the proposed model and a comparison to filtered backprojection is presented. In particular, we investigate how a reduction of the measurement data influences the image quality for a numerical phantom (Subsection 3.1) as well as for real data (Subsection 3.2). Finally, conclusions are drawn in Section 4.

## 2. Problem formulation and solution method

In photoacoustic tomography, a biological sample is excited by pulsed laser light, which is absorbed by natural endogenous chromophores or injected contrast agents. The absorbed energy is converted into ultrasound waves via the thermoelastic effect. Due to these ultrasound waves, an optical field outside of the sample attains a phase variation proportional to the pressure integrated along the probe beam path, which we measure using a CCD-camera resulting in a phase-contrast image. This phase-contrast image displays the integrated pressure induced phase variations:

(1) $$R[p_{T}](s,\varphi ,z)=\frac{2\pi }{\lambda_{PB}}\frac{dn}{dp}R[p_{T}](s,\varphi ,z)$$

with dn/dp the elasto-optic coupling coefficient, $\lambda_{PB}$ the wave length of the probe laser beam and $R[p_{T}]$ the two dimensional Radon transform of the pressure field p at the measurement time T defined by

(2) $$R[p_{T}](s,\varphi ,z):=\int_{-L}^{L}p_{T}(s\omega (\varphi )+r\omega^{⊥}(\varphi )+(0,0,z))\;dr.$$

Here, 2L is the integration length, ω(φ)=(cos⁡(φ),sin⁡(φ),0) and $\omega^{⊥}(\varphi )=(\sin(\varphi ),-\cos(\varphi ),0)$. The pressure $p_{0}$ at time 0 in the sample region is proportional to the absorbed energy. Hence, the task is to compute $p_{0}$ from $PI_{T}$ to obtain a 3D-reconstruction of the test sample where the light absorbing structures inside of the sample become visible.

The governing equation describing the pressure field p(t,x) at time t and location x=(x,y,z) is the wave equation

(3) $$\frac{\partial^{2}p}{\partial t^{2}}(t,x)=c_{s}^{2}\Delta p(t,x),$$

with initial pressure $p(0,x)=p_{0}(x)$, initial speed $\frac{\partial p}{\partial t}(0,x)=0$ and the speed of sound $c_{s}$. Combining this with the intertwining property of the Radon transform [49, Chapter 1, Lemma 2.1], i.e.,

(4) $$R[\Delta p](s,\varphi ,z)=\Delta_{s,z}R[p](s,\varphi ,z),$$

where $\Delta_{s,z}$ is the Laplace operator with respect to s and z, yields the governing equation of the projection data

(5) $$\frac{\partial^{2}}{\partial t^{2}}R[p](t,s,\varphi ,z)=c_{s}^{2}\Delta_{s,z}R[p](t,s,\varphi ,z),$$

with initial conditions $R[p](0,s,\varphi ,z)=R[p_{0}](s,\varphi ,z)$ and $\frac{\partial }{\partial t}R[p](0,s,\varphi ,z)=0$. Therefore, the initial pressure field $p_{0}$ can be recovered in two steps.

In the first step, the projections of the initial pressure distribution $R[p_{0}]$ have to be computed from the camera images $PI_{T}$. This can be done using (1) and back propagation in the frequency space [11,19]:

(6) $$f(s,\varphi ,z)\approx R[p_{0}](s,\varphi ,z)=F_{s,z}^{-1}\left[F_{s,z}\left[2R[p_{T}]\right]\;\cos(c_{s}|\cdot |T)\right](s,\varphi ,z),$$

where $F_{s,z}$ is the spatial Fourier operator with respect to s and z, and $\cos(c_{s}|\cdot |T)$ the time propagator in the frequency domain causing a forward and backward wave propagation. Note that $R[p_{T}]$ is given by $PI_{T}$ only in the field of view of the camera, which covers detection angles always smaller than $180^{\circ }$ of the acoustic wave patterns originating from structures inside the sample (Fig. 4(b)). This limited view problem introduces an additional error when applying Eq. (6) for the computation of $R[p_{0}]$ due to the lack of information along specific wave propagation directions [50]. This error affects the final imaging result independent from the second reconstruction step and is not subject of this work.

The second step is the reconstruction of the initial pressure $p_{0}$ from $f\approx R[p_{0}]$, which is the starting point of the proposed work. This problem, however, is ill-posed as the Radon transform is not continuously invertible on the data space, see, e.g., [51]. In practice, a filtered backprojection (FBP) algorithm is often used for the inversion of the Radon transform where appropriate filters have a regularizing effect, i.e., allow to overcome the ill-posedness. However, this approach requires the presence of the projection data for each line that passes the region of interest. The absence of full data, as it is the case if only a few projection angles are measured, typically leads to undesired streaking artifacts in the reconstructions. For this reason, we do not use FBP but consider instead the following minimization problem:

(7) $$\underset{p_{0}}{\text{min}}\;\frac{\mu }{2}{\left\|R[p_{0}]-f\right\|}^{2}+R_{\alpha }(p_{0}),$$

where $R_{\alpha }$ denotes a regularization term and α a possible parameter. One can think of $R_{\alpha }$ as a functional which penalizes unwanted features of a solution $p_{0}$. The first term ${\left\|R[p_{0}]-f\right\|}_{2}^{2}$ on the other hand ensures that the Radon transform of a solution $p_{0}$ of Eq. (7) is close to f. This approach is known in literature as Tikhonov regularization [21,52,53].

Previous works [47,48,54–56] suggest that Total Variation (TV) and Total Generalized Variation (TGV) are suitable candidates for the regularization term. In fact TV- or TGV-based regularization suppresses random noise and incoherent artifacts while preserving jumps in a solution. Let us give a definition of TV and TGV.

For a given smooth function p on a set Ω its Total Variation (TV) can be defined by

(8) $$\text{TV}(p)=\int_{\Omega }|\nabla p|dx.$$

While this representation is valid only for sufficiently smooth functions p for which the gradient and the integrals are well-defined, the Total Variation also makes sense for integrable functions whose distributional derivative is a Radon measure. We will, however, use TV only in a discrete setting where no problems arise from this definition.

The second-order Total Generalized Variation (TGV) of a smooth function p on Ω can be defined by

(9) $${\text{TGV}}_{\alpha }^{2}(p)=\underset{v}{\text{inf}}\;\alpha_{1}\int_{\Omega }|\nabla p-v|\;dx+\alpha_{0}\int_{\Omega }|E(v)|\;dx.$$

The infimum in this definition is taken over all vector fields v and E(v) denotes the symmetrized derivative $\frac{1}{2}(\nabla v+\nabla v^{\text{T}})$. The parameter $\alpha_{1}$ is typically set to 1 to allow for better comparability with TV, while a suitable choice of $\alpha_{0}$ is discussed in Section 3. Again, Definition (9) can be extended to non-smooth functions p, however, as before, we will only use it in the discrete setting.

Using TV as a regularizer in Tikhonov regularization for inverse problems eliminates fluctuations but enforces piecewise constant solutions. This, however, can lead to staircase artifacts in regions where the exact solution is not piecewise constant. By including not only first derivatives but also second derivatives TGV overcomes this particular issue, which is demonstrated for example in [36].

Using TGV as regularization term in (7) leads to the minimization problem

(10) $$\underset{p_{0}}{\text{min}}\;\frac{\mu }{2}{\left\|R[p_{0}]-f\right\|}^{2}+{\text{TGV}}_{\alpha }^{2}(p_{0}).$$

This is a non-smooth, convex minimization problem. In the following section we discuss how such a problem can be solved numerically.

### 2.1. Non-smooth, convex optimization

Problem (10) can be classified as a non-smooth, convex minimization problem. Hence, we would like to shortly discuss the mathematical background of how one can solve such problems in general [57,58]. For this purpose, let $H_{1}$ and $H_{2}$ be Hilbert spaces, $F:H_{1}\to \mathbb{R}$ be convex, proper and lower semi-continuous, i.e., there exists $u\in H_{1}$ such that F(u)<∞, for $u_{1}$, $u_{2}\in H_{1}$ and λ∈[0,1] there holds $F(\lambda u_{1}+(1-\lambda )u_{2})\leq \lambda F(u_{1})+(1-\lambda )F(u_{2})$, and $u_{n}\to u$ implies $F(u)\leq {\text{lim inf}}_{n}\;F(u_{n})$. Let furthermore $G:H_{2}\to {\mathbb{R}}^{\infty }$ be convex, proper and lower semi-continuous and $A:H_{1}\to H_{2}$ be linear and continuous. Consider the convex minimization problem:

(11) $$\underset{u\in H_{1}}{\text{min}}\;F(u)+G(Au).$$

Suppose that a minimum of this problem exists. Under suitable conditions (see, e.g., [58, Chapter III]), one can reformulate the minimization problem (11) to the saddle-point problem

(12) $$\underset{u\in \text{dom}\;F}{\text{min}}\underset{\xi \in \text{dom}\;G^{*}}{\text{sup}}L(u,\xi ),\;L(u,\xi ):=\langle \xi ,Au\rangle +F(u)-G^{*}(\xi ),$$

where $\text{dom}\;F=\{u\in H_{1}:F(u)\;<\;\infty \}$, and $G^{*}(\xi )$ is the Fenchel conjugate of G defined by

$$G^{*}\;(\xi )=\underset{u\in H}{\text{sup}}\;\langle \xi ,u\rangle -G(u),$$

and $\text{dom}\;G^{*}$ is given analogously to domF. In particular, if $\left(u^{*},\xi^{*}\right)$ is a solution of (12), then $u^{*}$ is a solution of (11). To solve the saddle-point problem (12), we use the primal-dual algorithm in [59], outlined in Algorithm 1, which is an iterative procedure.

**Algorithm 1:** Primal Dual Algorithm for the solution of (12)

**Require:** Parameters *σ*, *τ*>0, s.t. *στ*∥*A*∥^2^<1 **Initialize:** *u*^0^ = 0 ∊ *H*_1_, *ū*^0^ = 0 ∊ *H*_1_, *ξ*^0^ = 0 ∊ *H*_2_ **for** *n* = 0, 1, …, *N* – 1 **do** Dual Update: $\xi^{n+1}={\text{prox}}_{\sigma }^{G*}(\xi^{n}+\sigma A{\bar{u}}^{n})$ Primal Update: $u^{n+1}={\text{prox}}_{\tau }^{F}(u^{n}-\tau A^{*}\xi^{n+1})$ Extragradient update: *ū*^*n*+1^ = 2*u*^*n*+1^ − *u^n^* **end for** **return** (*u^N^*, *ξ^N^*)

Here, the *proximal mapping* ${\text{prox}}_{\tau }^{F}$ is defined by

$${\text{prox}}_{\sigma }^{F}(u_{0})=\underset{u\in H_{1}}{\text{arg min}}\frac{{\left\|u-u_{0}\right\|}^{2}}{2}+\sigma F(u),$$

and ${\text{prox}}_{\sigma }^{G^{*}}$ analogously. Run indefinitely, Algorithm 1 produces a sequence ${\left(u^{n},\xi^{n}\right)}_{n}$ (weakly) converging to a solution of (12). Here, we stop the computations after a predefined number of steps N, where N is large enough such that convergence can be observed, i.e., that $u^{N}$ is sufficiently close to a solution of (11).

### 2.2. Discretization and Radon inversion algorithm

To solve the minimization problem (10), we discretize it, bring it into the form of Eq. (11), and apply the primal-dual algorithm (Algorithm 1). Here we follow the lines of [60, Chapter V].

We start with the discretization by defining suitable discrete function spaces. By a scaling argument we can assume that the integration length L=1 and that the pressure fields are supported in the set $\Omega_{R}:=[-\frac{\sqrt{2}}{2},\frac{\sqrt{2}}{2}]\times [-\frac{\sqrt{2}}{2},\frac{\sqrt{2}}{2}]\times [0,h]$. We discretize this set $\Omega_{R}$ by subdividing it into $N_{x}\times N_{x}\times N_{z}$ equal cubes and describe these cubes by the index set $\left\{0,\ldots ,N_{x}-1\right\}\times \{0,\ldots ,N_{x}-1\}\times \{0,\ldots ,N_{z}-1\}$. On this set we can define the following function spaces:

(13) $$P:={\mathbb{R}}^{N_{x}\times N_{x}\times N_{z}},\;V:={\left({\mathbb{R}}^{3}\right)}^{N_{x}\times N_{x}\times N_{z}},\;W:={\left({\mathbb{R}}^{6}\right)}^{N_{x}\times N_{x}\times N_{z}}.$$

The elements in P represent functions which are constant on the above described cubes. This space P is going to be our reconstruction space. An element p∈P can be written in the form ${\left(p^{x,y,z}\right)}_{x=0,y=0,z=0}^{N_{x}-1,N_{x}-1,N_{z}-1}$, where $p^{x,y,z}$ is the value of p at the cube with index (x,y,z). Similarly, elements in V and W represent piecewise constant, vector-valued functions with 3 and 6 elements, respectively. These spaces contain the discrete gradients of functions in P and symmetrized gradients of functions in V needed for the application of TGV in the discrete setting.

For the discretization of the data space or sinogram space, i.e., the space of functions on the set $\Omega_{S}:=[-1,1]\times [0,\pi ]\times [0,h]$, we consider $S:={\left(\mathbb{R}\right)}^{N_{s}\times N_{\varphi }\times N_{z}}$. Here, $N_{s}$ denotes the number of parallel lines whose integrals are measured, and which is usually set to approximately $\sqrt{2N_{x}^{2}+2N_{y}^{2}}$. Related to the experimental setup, $N_{s}$ and $N_{z}$ denote the amount of pixels of the camera in horizontal and vertical direction. The value $N_{\varphi }$ denotes the number of angles from which projections are taken. For the sake of simplicity, we assume in this paper that uniformly distributed angles in the interval [0,π) are used. Non-uniform choices are also possible within the presented framework when taking straightforward adaptations into account, and might lead to further improvements in reconstruction quality [32].

The spaces P and S equipped with the respective norms

$${\left\|p\right\|}_{P}^{2}=\sum_{x=0}^{N_{x}-1}\sum_{y=0}^{N_{x}-1}\sum_{z=0}^{N_{z}-1}{\left|p^{x,y,z}\right|}^{2},\;{\left\|f\right\|}_{S}^{2}=\sum_{s=0}^{N_{s}-1}\sum_{\varphi =0}^{N_{\varphi }-1}\sum_{z=0}^{N_{z}-1}{\left|f^{s,\varphi ,z}\right|}^{2},$$

are Hilbert spaces. The corresponding inner products in both spaces are the sums of the elementwise products. Later we will omit the indices P and S of the norms when it is clear from the context which norm is used.

Using these spaces we can now consider the discrete minimization problem

(14) $$\underset{p\in P}{\text{min}}\frac{\mu }{2}{\left\|Rp-f\right\|}^{2}+{\text{TGV}}_{\alpha }^{2}(p),$$

where p and f denote the discrete solution and discrete datum, and R and ${\text{TGV}}_{\alpha }^{2}$ the discrete Radon transform and discrete TGV, respectively. For details about the regularization with αTV instead of ${\text{TGV}}_{\alpha }^{2}$, we refer to Appendix A.2. The discretization of the Radon transform and its adjoint, which is needed later, is a little cumbersome. We use the discretization described in [60, Chapter V] to which we refer regarding further details. Here we continue by defining a suitable discretization of TGV.

For sufficiently smooth functions the integrals in the definition of TGV (cf. Eq. (9)) can be understood as the $L^{1}$-norms of the Euclidean norms of the respective vector-valued functions. Hence, for functions v∈V we can discretize such integrals by the $\ell^{1}$ norm

(15) $${\left\|v\right\|}_{\ell^{1}}:=\sum_{x=0}^{N_{x}-1}\sum_{y=0}^{N_{x}-1}\sum_{z=0}^{N_{z}-1}|v^{x,y,z}|,$$

and for functions w∈W by the analogously defined norm ${\left\|w\right\|}_{\ell^{1}}$. Note that an element in ${\mathbb{R}}^{6}$ is interpreted as a symmetric matrix, and hence for such elements, |⋅| denotes the Frobenius norm. This leads to the definition

(16) $${\text{TGV}}_{\alpha }^{2}(p)=\underset{q\in V}{\text{min}}\;\alpha_{1}{\left\|\nabla p-q\right\|}_{\ell^{1}}+\alpha_{0}{\left\|Eq\right\|}_{\ell^{1}}\;\text{ for all }p\in P.$$

Here, ∇ and E denote discrete versions of the gradient and the symmetrized derivative. They can be implemented using a standard finite difference approach, which is described in detail in Appendix A.1. By inserting this definition, we can rewrite the minimization problem (14) to

(17) $$\underset{p\in P,q\in V}{\text{min}}\frac{\mu }{2}{\left\|Rp-f\right\|}^{2}+\alpha_{1}{\left\|\nabla p-q\right\|}_{\ell^{1}}+\alpha_{0}{\left\|Eq\right\|}_{\ell^{1}}.$$

Next, we would like to bring this minimization problem into the form (11) described in Section 2.1. For this purpose, we define the spaces $H_{1}:=P\times V$, $H_{2}:=S\times V\times W$ and the operators $F:H_{1}\to {\mathbb{R}}^{\infty }$ and $G:H_{2}\to {\mathbb{R}}^{\infty }$ by

(18) $$F(p,q):=0,\;G(g,v,w):=\frac{\mu }{2}{\left\|g-f\right\|}^{2}+\alpha_{1}{\left\|v\right\|}_{\ell^{1}}+\alpha_{0}{\left\|w\right\|}_{\ell^{1}}.$$

Furthermore, we define the linear operator $A:H_{1}\to H_{2}$ by

(19) A(p,q):=(Rp,∇p−q,Eq).

With these definitions, the minimization problem (17) can indeed be written in the form ${\text{min}}_{p,q}\;F(p,q)+G(A(p,q))$ and the theory in Section 2.1 can be shown to apply. In particular, we can use Algorithm 1 to solve it. For this purpose, we need to compute ‖A‖ and construct ${\text{prox}}_{\sigma }^{G^{*}}$, ${\text{prox}}_{\tau }^{F}$ and $A^{*}$.

The adjoint operator $A^{*}:H_{2}\to H_{1}$ is given by

(20) $$A^{*}(g,v,w)=(R^{*}g-\mathrm{div}v,-\mathrm{div}w-v).$$

The operator $R^{*}$ in this definition is the adjoint of R and div denotes the discrete divergence on V and W, respectively. Again we refer to Section A.1 for a detailed description of the discrete differential operators div on the respective spaces.

For the computation of ‖A‖ we assume without loss of generality that ‖R‖≤1. Indeed, we can check this assumption by approximating ‖R‖ by applying the power iteration [61,62] to approximate the largest eigenvalue λ of $R^{*}R$. If λ>1 and hence ‖R‖>1 we can scale the operator R, its adjoint $R^{*}$ and the discrete datum f by a factor 1/λ and end up with a new operator R which satisfies ‖R‖≤1. Under this assumption, one can show [38, cf. Section 3.2] that ${\left\|A\right\|}^{2}<\frac{\sqrt{32}}{2}+9<12$.

In our application, we have F≡0. Therefore, it is easy to see that the proximal mapping ${\text{prox}}_{\tau }^{F}$ is given by the identity on $H_{1}$, i.e.,

$${\text{prox}}_{\tau }^{F}(p,q)=(p,q),$$

independently of τ.

To compute the proximal mapping ${\text{prox}}_{\sigma }^{G^{*}}$ we first have to compute the Fenchel conjugate $G^{*}$. Due to the fact that the three summands in the definition of G all depend on a different variable of the product space $H_{2}$, it follows

$$G^{*}(g,v,w)={\left(\frac{\mu }{2}{\left\|\cdot -f\right\|}^{2}\right)}^{*}(g)+{\left(\alpha_{1}{\left\|\cdot \right\|}_{\ell^{1}}\right)}^{*}(v)+{\left(\alpha_{0}{\left\|\cdot \right\|}_{\ell^{1}}\right)}^{*}(w).$$

One can show [63] that in general, there holds ${\left({\left\|\cdot \right\|}_{H}\right)}^{*}(\zeta )=\chi_{\left\{{\left\|\cdot \right\|}_{H}^{*}\leq 1\right\}}(\zeta )$, where

$$\chi_{B}(\zeta )=\left\{ \begin{array}{ll} 0, & \text{if }\zeta \in B,\\ \infty , & \text{otherwise}, \end{array}\right.$$

and ${\left\|\cdot \right\|}_{H}^{*}$ is the dual norm of ${\left\|\cdot \right\|}_{H}$. Furthermore, for a general F and α>0, it holds that ${\left(\alpha F\right)}^{*}(\zeta )=\alpha F^{*}(\zeta /\alpha )$. Hence, we get

$${\left(\alpha_{1}{\left\|\cdot \right\|}_{\ell^{1}}\right)}^{*}(v)=\chi_{\left\{{\left\|\cdot \right\|}_{\ell^{\infty }}\leq \alpha_{1}\right\}}(v),\;{\left(\alpha_{0}{\left\|\cdot \right\|}_{\ell^{1}}\right)}^{*}(w)=\chi_{\left\{{\left\|\cdot \right\|}_{\ell^{\infty }}\leq \alpha_{0}\right\}}(w),$$

where ${\left\|v\right\|}_{\ell^{\infty }}=\text{max}\{|v^{x,y,z}|:0\leq x,y<N_{x},0\leq z\;<\;N_{z}\}$ for v∈V and analogously for w∈W. Finally, computations yield

$${\left(\frac{\mu }{2}{\left\|\cdot -f\right\|}^{2}\right)}^{*}(g)=\frac{1}{2\mu }{\left\|g\right\|}^{2}+\langle g,f\rangle .$$

We are now ready to compute ${\text{prox}}_{\sigma }^{G^{*}}$. By the same argument as before, we can compute the proximal mappings of all of the summands of $G^{*}$ independently and get

(21) $${\text{prox}}_{\sigma }^{G^{*}}(g,v,w)=\left({\text{prox}}_{\sigma }^{{\left((\mu /2){\left\|\cdot -f\right\|}^{2}\right)}^{*}}(g),\;{\text{prox}}_{\sigma }^{{\left(\alpha_{1}{\left\|\cdot \right\|}_{\ell^{1}}\right)}^{*}}(v),\;{\text{prox}}_{\sigma }^{{\left(\alpha_{0}{\left\|\cdot \right\|}_{\ell^{1}}\right)}^{*}}(w)\right).$$

One can show that

(22) $${\text{prox}}_{\sigma }^{{\left((\mu /2){\left\|\cdot -f\right\|}^{2}\right)}^{*}}(g)=\frac{\mu }{\mu +\sigma }(g-\sigma f),$$

while the two remaining proximal mappings correspond to the projections

(23) $${\text{prox}}_{\sigma }^{{\left(\alpha_{1}{\left\|\cdot \right\|}_{\ell^{1}}\right)}^{*}}(v)={\text{proj}}_{\left\{\bar{v}\in V:\;{\left\|\bar{v}\right\|}_{\ell^{\infty }}\leq \alpha_{1}\right\}}(v),$$

(24) $${\text{prox}}_{\sigma }^{{\left(\alpha_{0}{\left\|\cdot \right\|}_{\ell^{1}}\right)}^{*}}(w)={\text{proj}}_{\left\{\bar{w}\in W:\;{\left\|\bar{w}\right\|}_{\ell^{\infty }}\leq \alpha_{0}\right\}}(w),$$

where

(25) $${\left({\text{proj}}_{\left\{\bar{v}\in V:\;{\left\|\bar{v}\right\|}_{\ell^{\infty }}\leq \alpha_{1}\right\}}(v)\right)}^{x,y,z}=\left\{ \begin{array}{ll} v^{x,y,z} & \text{if }|v^{x,y,z}|\leq \alpha_{1},\\ \frac{\alpha_{1}}{|v^{x,y,z}|}v^{x,y,z} & \text{if }|v^{x,y,z}|\;>\;\alpha_{1}, \end{array}\right.$$

and ${\text{proj}}_{\left\{\bar{w}\in W:\;{\left\|\bar{w}\right\|}_{\ell^{\infty }}\leq \alpha_{0}\right\}}(w)$ is defined analogously.

**Algorithm 2:** Primal Dual Algorithm for the solution of (14)

Estimate ∥**R**∥ using the power iteration on R*R and scale **R**, R* and *f* by 1/∥**R**∥. **Require:** Parameters *σ*, *τ*>0, s.t. $\sigma \tau <\frac{1}{12}$ **Initialize:** *p*^0^ = 0, ${\bar{p}}^{0}=0\;\text{in}\;P$, *q*^0^ = 0, ${\bar{q}}^{0}=0$, *v*^0^ = 0 in *V*, *w*^0^ = 0 in *W*, *g*^0^ = 0 in *S* **for** *n* = 0, 1, …, *N* – 1 **do** Dual Update: $$\begin{array}{l} g^{n+1}=\frac{\mu }{\sigma +\mu }(g^{n}+\sigma (R\;{\bar{p}}^{n}-f))\\ v^{n+1}={\text{proj}}_{\left\{\bar{v}\in V:\;{\left\|\bar{v}\right\|}_{\ell^{\infty }}\leq \alpha_{1}\right\}}(v^{n}+\sigma (\nabla {\bar{p}}^{n}-{\bar{q}}^{n}))\\ w^{n+1}={\text{proj}}_{\left\{\bar{w}\in W:\;{\left\|\bar{w}\right\|}_{\ell^{\infty }}\leq \alpha_{0}\right\}}(w^{n}+\sigma E{\bar{q}}^{n}) \end{array}$$ Primal Update: $$\begin{array}{l} p^{n+1}=p^{n}-\tau (R^{*}g^{n+1}-\text{div}\;v^{n+1})\\ q^{n+1}=q^{n}+\tau (v^{n+1}+\text{div}\;w^{n+1}) \end{array}$$ Extragradient Update: $$\begin{array}{l} {\bar{p}}^{n+1}=2p^{n+1}-p^{n}\\ {\bar{q}}^{n+1}=2q^{n+1}-q^{n} \end{array}$$ **end for** **return** *p^N^*

With this knowledge, one can now apply Algorithm 1 for the solution of the discrete minimization problem (14), which yields Algorithm 2. For sufficiently large N the result $p^{N}$ can be regarded as a solution of (14).

## 3. Experiments and results

To evaluate our PAT reconstruction method we test it using purely numerical data as well as given real-life data from previous experiments. In particular, we examine how a reduction of the number of projection angles influences the reconstruction quality. We proceed as described in Section 2. by first recovering $R[p_{0}]$ via Eq. (6) and then inverting the Radon transform via Tikhonov regularization with TGV (or TV) as regularization term by solving (10) via Algorithm 2. For the latter we use the software tool Graptor [64], which provides a powerful OpenCL/GPU implementation that we slightly adapt to allow negative values in the solutions.

In all numerical computations, the number of iterations N in Algorithm 2 is chosen such that for larger choices, no significant changes in the reconstructed images are noticeable. In particular, the actual choice of N generally overestimates the number of iterations needed for obtaining the reconstruction. This way, we ensure that no artifacts or distortions originating from early stopping appear in the reconstructions. On the other hand, the computation times of Algorithm 2 essentially depend on the number of iterations, which is why in practice, one should choose N as small as possible. The difficulty is that the required number of iterations depends on the considered data and the chosen parameters, in particular on the regularization parameter μ. Nevertheless, the iterative nature of Algorithm 2 allows to consider intermediate reconstruction results on the fly, and to stop when these results are satisfactory.

Let us further comment on the choice of the regularization parameter μ in Eq. (7) which acts as a weighting factor in the minimization problem. If μ is chosen large, then the discrepancy term ${\left\|R[p_{0}]-f\right\|}^{2}$ in Eq. (7) dominates and the impact of the regularization term is small. This is reasonable in case of low noise levels, while in case of high noise levels, μ should be chosen small. However, a good choice of μ is not known a priori and, in particular, depends on the considered data. This is why we use various values of μ in the computations and finally choose the best parameter for each datum manually by a visual comparison of the resulting reconstructions, selecting the parameter for which obvious artifacts are eliminated or reduced while at the same time, desired features are sharply visible and not blurred. We emphasize that this approach is also feasible when no ground truth image is available, as it is the case in the experimental tests in Section 3.2.

For all computations with TGV as a regularization term we use the parameters $\alpha_{1}=1$ and $\alpha_{0}=2.5$ (cf. Eq. (9)). The choice of $\alpha_{1}$ allows for a better comparability with TV as mentioned before, while the choice of $\alpha_{0}$ is the default choice in the software tool Graptor [64] and is used since it yields satisfactory results in all presented computations.

All computations are executed on a work station with an Nvidia Tesla 40K GPU, which features 12GB of GDDR5 RAM and 2880 CUDA cores with 745 MHz clock rate. For comparison we compute the inverse Radon transform also with a filtered back projection algorithm using the Matlab routine iradon [65].

### 3.1. Numerical phantom tests

**Fig. 1.**
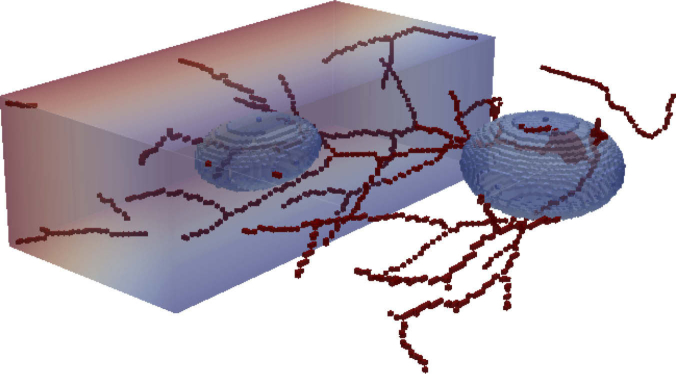
3D model of synthetic pressure distribution.

We start with a purely numerical test to compare the different methods for the inversion of the Radon transform. For this purpose we take a vessel data set of size 128×128×128 voxels which we reduce to a skeleton using the code in [66]. The pressure of this structure is set to 1. We add two ellipsoids with variable pressure and a ramp with linearly increasing pressure to this pressure field. Finally, we select 40 slices resulting in a pressure distribution of size 128×128×40, which we embed in a larger domain for the computations. Overall, the composed phantom contains typical PAT structures representing the pressure distribution of absorbing vasculature, tumor structures and tissue background visualized in Fig. 1

.

**Fig. 2.**
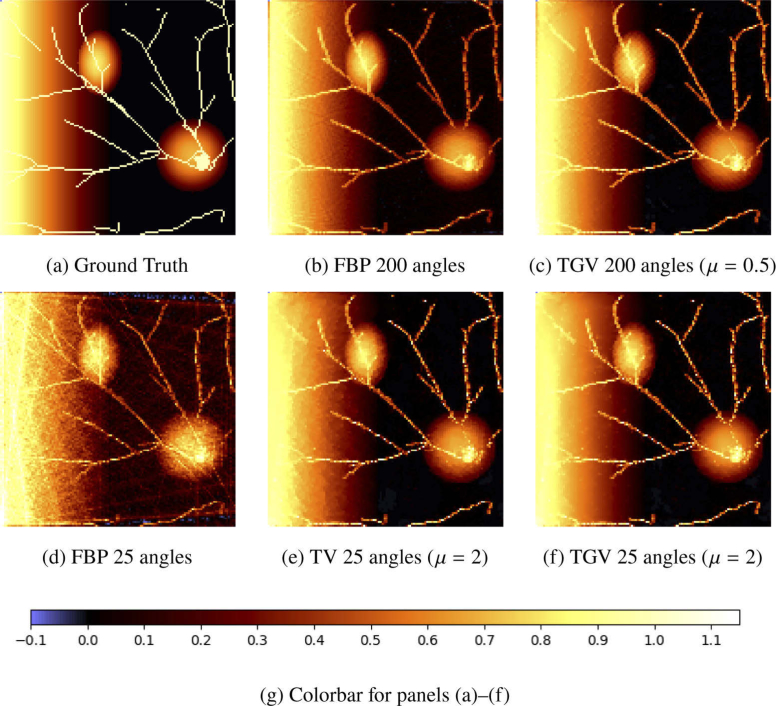
(a)–(f) Maximum amplitude projections along the z axis of the reconstructions of numerical phantom data containing some thin vessel-like structures, two ellipsoids and a ramp. The reconstruction method is indicated below each image, together with the choice of the regularization parameter μ. All images share the same colormap, see (g).

We simulate the measurement process for this initial distribution for 200 and 25 equidistant angles, respectively, ending up with phase-contrast data $PI_{T}$ (cf. Eq. (1)). Here, we choose the field of view of the camera so large that it covers the whole wave pattern of the pressure field $p_{T}$ at measurement time T. This reduces the error of the first reconstruction step according to Eq. (6) and allows for a better comparison of the different methods for the inversion of the Radon transform. For the latter, the number N of iterations in Algorithm 2 is chosen to be 10000 in case of 200 angles, and 20000 in case of 25 angles. The results of our computations are presented in Figs. 2

and 3

**Fig. 3.**
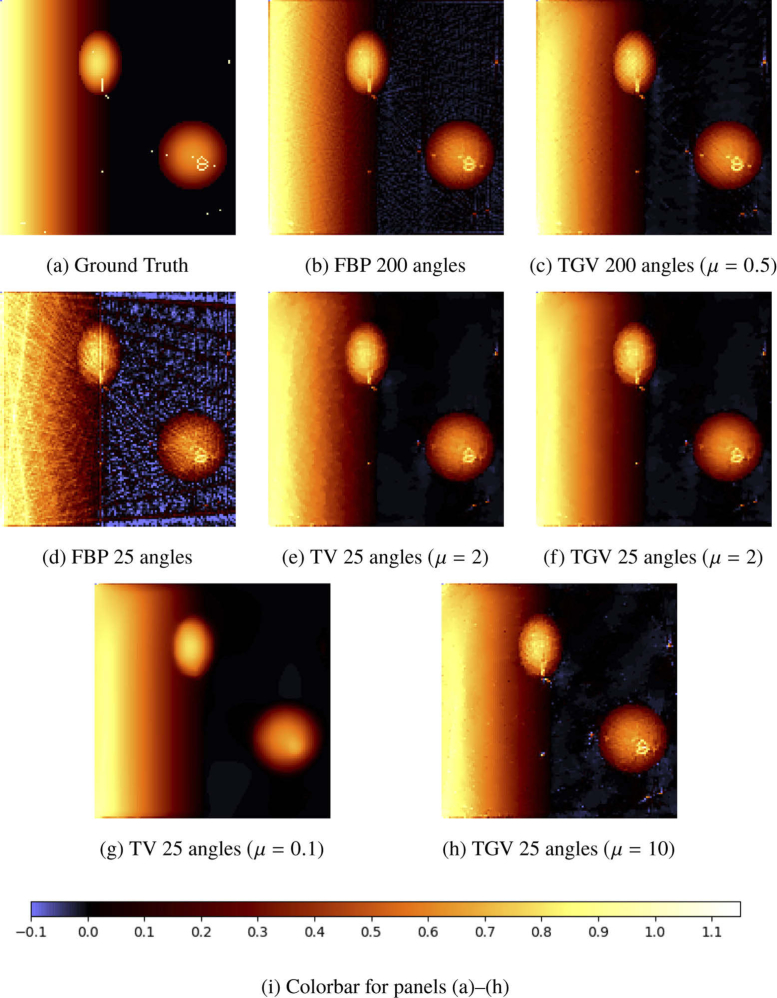
(a)–(h) Single slice of the reconstructions of numerical phantom data containing some thin vessel-like structures, two ellipsoids and a ramp. The reconstruction method is indicated below each image, together with the choice of the regularization parameter μ. All images share the same colormap, see (i).

below. Note that we restrict them to the original size of 128×128×40 for better comparability, while the reconstruction space P in the computations has the dimensions 281×281×40.

The corresponding computation times for the considered Radon transform inversion methods with either 200 or 25 angles can be found in Table 1

**Table 1.** Computation times for direct and variational Radon transform inversion of numerical phantom data containing some thin vessel-like structures, two ellipsoids and a ramp. The size of the computation domain is 281×281×40 voxels.

reconstruction method	computation time [s]
FBP, 200 angles	11.9
TV, 200 angles, 10000 iterations	1011.4
TGV, 200 angles, 10000 iterations	1056.8
FBP, 25 angles	8.2
TV, 25 angles, 20000 iterations	359.4
TGV, 25 angles, 20000 iterations	451.4

. Since it is a direct method, the computation times for FBP inversion are significantly lower than the times for the TV- and TGV-based inversion via an iterative procedure, where TGV only needs slightly more computation time in comparison to TV. Note that in the actual experiment, we observe sufficient convergence of the algorithm after 50% of the iterations, such that reconstruction times can easily be halved without compromising reconstruction quality. The time for the first inversion step (Eq. (6)) is not included in the values in Table 1. It depends on the number of used angles and is about 2.07s in case of 200 angles and 0.26s in case of 25 angles.

A popular way to present results of 3D pressure distributions is to display the maximum amplitude projections (MAP) along the three coordinate axes. This representation is frequently used due to the better appearance of imaging results but not always useful for clinical purposes where depth information is a necessity. Exemplary, we present the maximum amplitude projections of the various reconstructions along the z-axis in Fig. 2.

The pressure in all reconstructions exceeds the interval [0,1] in contrast to the ground truth image, which is why we choose the interval [−0.1,1.15] for the colormap in our visualization. In the first line of Fig. 2 the ground truth image (a) as well as the FBP (b) and TGV solution (c) for 200 angles are given. We do not present the maximum amplitude projection of the TV solution for 200 angles here because it is optically not distinguishable from the TGV solution for 200 angles. All reconstructions using 200 angles yield reasonable results. The thin structures appear to be brighter in the TGV reconstruction. However, they do not have a constant pressure equal to 1 neither in the FBP nor in the TGV solution. This is visible in the lower right circle, where one has difficulties to see the knot-like structure formed by the vessel structure, which is clearly recognizable in the ground truth solution.

In the second line of Fig. 2 the FBP (d), TV (e) and TGV (f) solutions for 25 angles are given. We have chosen this low number of angles to demonstrate the benefits of TV- and TGV-regularized Radon inversion over the FBP. While the FBP solution is full of stripe-like artifacts, we can recover the images quite well when using TV or TGV regularization. The TGV solution (f) is also free from staircase artifacts, which can be seen for example on the ramp in (e) and are typical for TV regularization. However, some thin structures disappeared or lost their intensity in all reconstructions from a reduced number of angles. In particular, some structures lying inside of the ellipsoids and the ramp, where the surrounding pressure is comparably high, are affected. Let us discuss this phenomenon further considering a certain slice of the 3D pressure distribution.

In Fig. 3, we extract a single slice of the 3D pressure distributions shown in Fig. 2. All phenomena described for Fig. 2 are again visible, in particular, the quality gain using variational reconstruction with TV and TGV. Nevertheless, we notice in the first row that the reconstruction quality already slightly suffers for 200 angles in those parts of the solutions with high background pressures, for example in the upper left ellipse. As it can be expected, quality becomes worse for reconstruction from 25 angles. The isolated point in the upper left ellipse for example, which is clearly visible in the ground truth image, cannot be identified in the solutions (d) to (f). Also, other structures suffer from reduced brightness. This explains why some structures are missing in the maximum amplitude projections.

Fig. 3 contains an additional row in which the effects of inappropriate choices of the regularization parameter are observable. In Fig. 3(g), the parameter μ is chosen too small. Therefore, the regularization term dominates and enforces high smoothness of the solution, such that the thin structures cannot be recovered. On the other hand, if μ is chosen too large, then the effect of the regularization is too weak and some artifacts can persist in the reconstruction, as can be seen in Fig. 3(h).

Let us point out that for this data set as well as the data sets described below, it turns out that the regularization parameter μ needs to be chosen in dependence on the number of projections for the measured data in addition to its signal-to-noise ratio (SNR), with a greater regularization parameter for less projections. We therefore expect that for fixed SNR, a parameter learning approach with respect to the model $\mu (N_{\varphi })=\mu_{0}+\mu_{1}N_{\varphi }^{-1}$ where $\mu_{0}\;>\;0$ and $\mu_{1}\;>\;0$ and $N_{\varphi }$ is the number of measured projections would yield a practical regularization parameter estimate. Such an approach has, for instance, successfully been carried out for TGV regularization in the context of magnetic resonance imaging (MRI) [44], where an affine-linear parameter model in dependence on the undersampling factor turned out to yield meaningful regularization parameters. As for radial undersampling in MRI, which exactly corresponds to few-angle tomography, the undersampling factor is proportional to the reciprocal of the measured projections, we expect that a straightforward adaptation would also work in the context of PAT imaging.

Finally, we report the peak signal-to-noise ratio (PSNR) and the structural similarity index (SSIM) for the different reconstruction methods with respect to the ground truth in Table 2

**Table 2.** PSNR and SSIM values for the reconstructions obtained from numerical phantom data containing some thin vessel-like structures, two ellipsoids and a ramp, with respect to the ground truth.

reconstruction method	PSNR [dB]	SSIM
FBP, 200 angles	31.06	0.921
TV, 200 angles, μ=0.5	30.58	0.945
TGV, 200 angles, μ=0.5	30.68	0.949
FBP, 25 angles	25.06	0.549
TV, 25 angles, μ=2	30.39	0.937
TGV, 25 angles, μ=2	30.28	0.943

to quantify our results. Both values are commonly used to measure the similarity of two images, with high PSNR/SSIM being better and SSIM ranging from 0 to 1. The results in Table 2 confirm the previous observations. In case that 200 angles are used for the reconstruction, all methods yield good results, which is what we also observed in Fig. 3. The results for the variational reconstruction methods are slightly worse according to PSNR and slightly better according to SSIM. In contrast, in case that only 25 angles are used, the PSNR and SSIM values decrease drastically for the FBP reconstruction, while they do not change significantly for the TV and TGV reconstructions.

In summary, we see that the variational reconstruction methods using TV and TGV as regularizers perform well for the considered numerical phantom. In particular we see that the number of angles can be reduced without introducing additional artifacts in the resulting images. However, we also observe that certain small structures can be lost or suffer from reduced brightness when the number of angles is low. Therefore, the number of angles should not be reduced too much in general.

### 3.2. Experimental tests

**Fig. 4.**
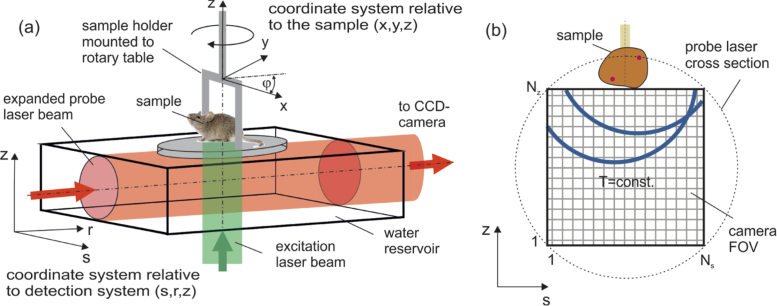
(a) Schematic showing the coordinate systems relative to the sample (x,y,z) and relative to the experimental setup (s,r,z). During wave pattern image acquisition the sample is rotated by an angle φ about the z-axis. (b) Image acquisition with the camera-based setup. The sample is located above the field of view (FOV) of the camera. The data structure is a snap-shot of the acoustic field at time T and sample orientation φ.

In this subsection we consider real-life data acquired in previous experiments. The data was gathered with a camera based ultrasound detection method introduced by Nuster et al. [11]. For the acquisition of the projection data from many directions the sample is centered in a sample holder with a center hole, slightly dipped in water for acoustic coupling and mounted on a rotary table. Pulsed laser light with 532 *nm* wavelength is used to illuminate the sample from below to excite the acoustic transients propagating out of the sample towards a volume accessible by the probe beam (Fig. 4(a)

). Subsequently snap-shots of the acoustic waves are recorded from different projection angles φ of the sample with a CCD-camera (Fig. 4(b)). The timing is achieved using a probe laser pulse to exposure the camera after a specified delay T with respect to the excitation laser pulse, which was 7μs for the phantom and 9μs for the in-vivo experiment.

The considered data sets, from phantom and in-vivo experiments, contain 200 and 100 projection images, respectively, which are homogeneously distributed over 180°. The phantom data sets were recorded with the smallest experimentally adjustable angular step size of 0.9°, and the in-vivo data sets with an angular step size of 1.8°. Hence, the obtainable 3D imaging resolution $\Lambda^{min}$ should be 100 μm for the phantom data and 200 μm for the mouse data in a cube imaging volume with 10 mm side length SL regarding the relation $\Delta \varphi^{max}=90^{\circ }\Lambda^{min}/SL$, derived from the Fourier-Slice-Theorem and the Nyquist-criterion [67,68].

The reconstruction of the initial pressure $p_{0}$ from the data sets is done again by back propagation of the projection data (Eq. (6)) and the inversion of the Radon transform by either a filtered back projection algorithm or by Algorithm 2. We compare the results of the reconstruction using once all 200/100 and once only 50/25 angles and stop Algorithm 2 after 5000 and 10000 iterations, respectively. Results for the TV-regularized inversion are not shown, because for the two specific experiments described below, we always attained, with TGV-regularized inversion, at least the quality of TV-regularized inversion.

#### 3.2.1. Phantom sample

We consider first a phantom sample. This sample was made of three black human hair loops with diameters ranging from 55 to 70 μm and black polystyrene microspheres with diameters 110 μm embedded in a mixture of 2% agar and 5% intralipid in water to mimic optical scattering properties of biological tissue. Accordingly, in case of ideal conditions it should be able to clearly identify loop shaped structures and bright spots in maximum amplitude projection (MAP) images along z-direction from 3D reconstructions.

We start by reporting the computation times in Table 3

**Table 3.** Computation times for direct and variational Radon transform inversion of phantom sample data containing black human hair loops and black polystyrene microspheres. The reconstruction space P has the dimensions 571×571×91.

reconstruction method	computation time [s]
FBP, 200 angles	4.0
TGV, 200 angles, 5000 iterations	5449.7
FBP, 50 angles	1.5
TGV, 50 angles, 10000 iterations	3420.3

. Again, the times for the FBP-inversion are significantly lower than for the TGV-regularized inversion. All times are higher than the ones reported in Table 1 which is due to the larger reconstruction space P that consists of 571×571×91 voxels for the experimental phantom sample.

In Fig. 5

**Fig. 5.**
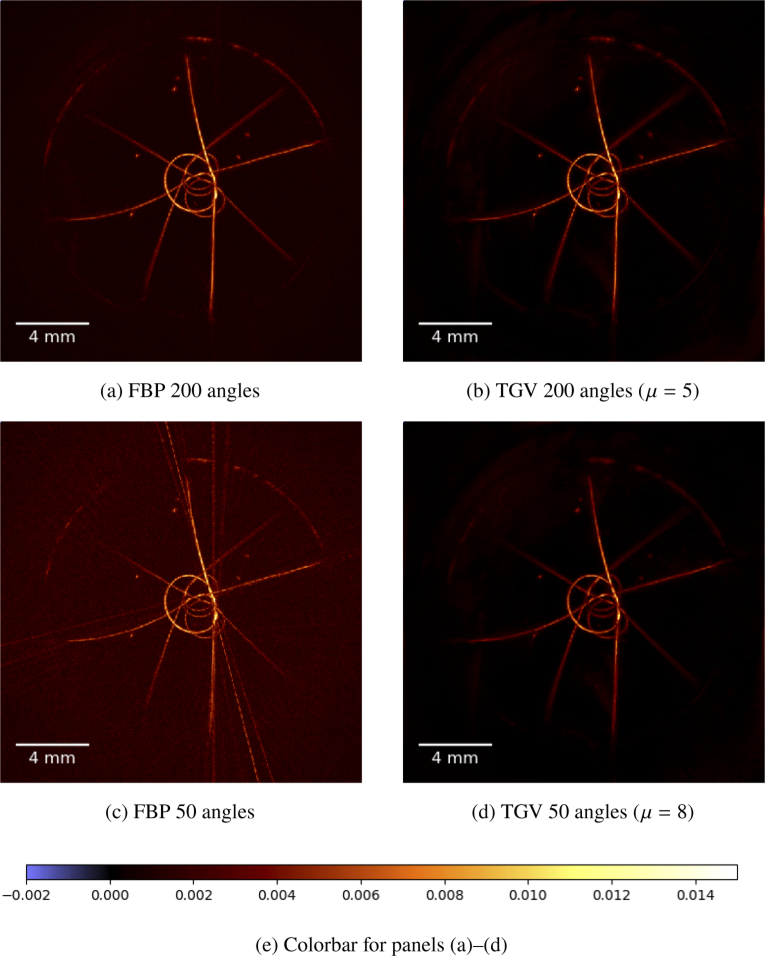
(a)–(d) Maximum amplitude projections along the z-axis of the phantom sample containing black human hair loops and black polystyrene microspheres. The reconstruction method is indicated below each image, together with the choice of the regularization parameter μ. All images share the same colormap, see (e).

we present the maximum amplitude projection images along the z-axis for 200 and 50 angles, respectively. The results for 200 angles appear reasonable for the FBP reconstruction in (a) as well as for the TGV reconstruction in (b). If the number of angles is reduced to 50 we can see prominent stripe artifacts appearing in the MAP image in (c) for the FBP reconstruction. Such artifacts are expected because the sampling criterion with 3.6° angular step size is obviously violated for the hair structures with diameters smaller than 400 μm. The TGV result in (d) clearly shows the advantage of variational regularization methods compared to the standard FBP, since even image reconstruction with highly undersampled angular data provides reasonable image quality without stripe artifacts.

In Fig. 6

**Fig. 6.**
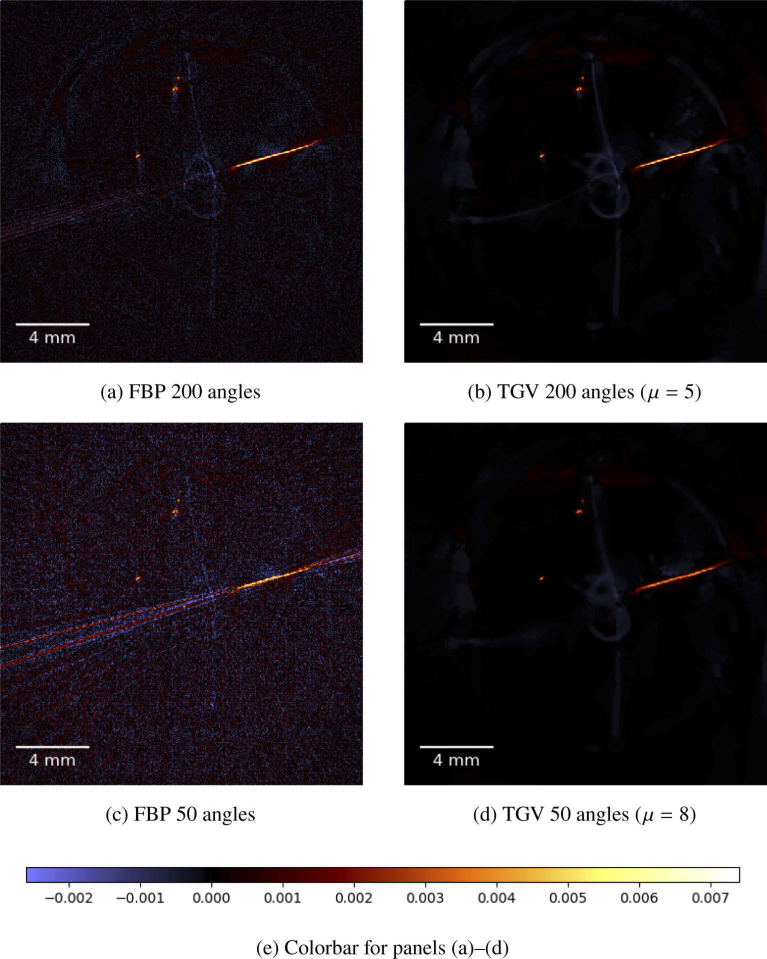
(a)–(d) Single slice of the phantom sample containing black human hair loops and black polystyrene microspheres. The reconstruction method is indicated below each image, together with the choice of the regularization parameter μ. All images share the same colormap, see (e).

, a single slice of the different solutions is presented. Here we can see that even in case of 200 angles, the FBP solution suffers from noise and small artifacts. In particular, we notice a blue, loop-like structure in the center of the image in Fig. 6(a). This does not directly correspond to a hair loop in the sample but is a ghost artifact that presumably stems from the first inversion step (cf. Eq. (6)). The noise and the small artifacts are no longer visible in the TGV solution in (b). The loop-like artifact is reduced, but still present in the center of the reconstructed image in form of a blurry structure. All other features are preserved in the TGV reconstruction. When reducing the number of angles, we can clearly see the stripe artifacts in the FBP solution, which seem to be caused by the long straight part of the hair in this slice. Furthermore, we notice increased noise artifacts in comparison to the 200 angles FBP solution which cause, in particular, the two points in the center (which correspond to hair segments orthogonally passing through the slice) being buried under noise. The TGV solution in Fig. 6(d) is able to eliminate both the noise and the stripe artifacts. The loop-like artifact in the center is less pronounced than in Fig. 6(b), but still observable as a strongly blurred structure. However, the two points in the center are barely visible anymore. In summary, the TGV reconstruction for 50 angles significantly removes reconstruction artifacts while preserving essentially all features that are recognizable in the FBP reconstruction.

**Fig. 7.**
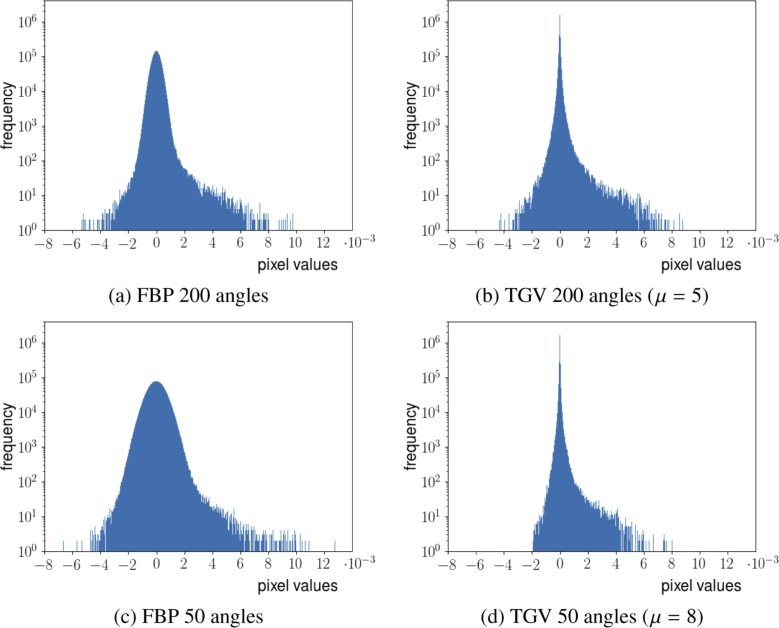
(a)–(d) Histograms of the 3D reconstructions of the phantom sample containing black hair loops and black polystyrene microspheres. The reconstruction method is indicated below each image, together with the choice of the regularization parameter μ.

With a ground truth reconstruction being unavailable for this data set, it is generally hard to quantify reconstruction quality. One approach that nevertheless yields some quantitative information is a comparison of the histograms of the reconstructed 3D images for the phantom sample, see Fig. 7

. For this sample, the majority of the reconstructed image can be expected to be constant zero, and the non-zero values are either caused by reconstruction artifacts or by the hair loops and microspheres. Neglecting the latter, the histogram around zero can therefore provide quantitative information about these artifacts. Having this in mind, one can clearly see that in case of TGV reconstruction, the peak of the histograms is rather centered around zero while in case of FBP reconstruction, the peak is less localized and resembles more a normal bell curve, which can be interpreted as the TGV reconstruction having less artifacts than the FBP reconstruction. Also, computing the mean values and the standard deviations of the various 3D-reconstructions confirms this interpretation. Note that for the computation of these values we consider all pixels, in particular non-zero pixels corresponding to the features of interest, i.e. the hair loops and the microspheres. This introduces a bias in the mean value and the standard deviation which is, however, as mentioned above, small since the ratio of these features and the zero background is negligibly low. In particular, no segmentation of the data is necessary for the computations. The resulting mean values and standard deviations for the different reconstructions are given in Table 4

**Table 4.** Mean value and standard deviations for various reconstructions of the phantom sample.

reconstruction method	mean value	standard deviation
FBP, 200 angles	$-9.65\cdot 10^{-8}$	$3.38\cdot 10^{-4}$
TGV, 200 angles, μ=5	$4.25\cdot 10^{-7}$	$1.39\cdot 10^{-4}$
FBP, 50 angles	$-5.53\cdot 10^{-7}$	$6.34\cdot 10^{-4}$
TGV, 50 angles, μ=8	$1.94\cdot 10^{-7}$	$1.12\cdot 10^{-4}$

. While the various mean values are reasonably close to zero, the standard deviations for TGV reconstructed images are considerably lower than for FBP reconstructed images, which quantitatively confirms the reduction of reconstruction artifacts provided by TGV regularization for this experiment.

#### 3.2.2. In vivo measurements

**Fig. 8.**
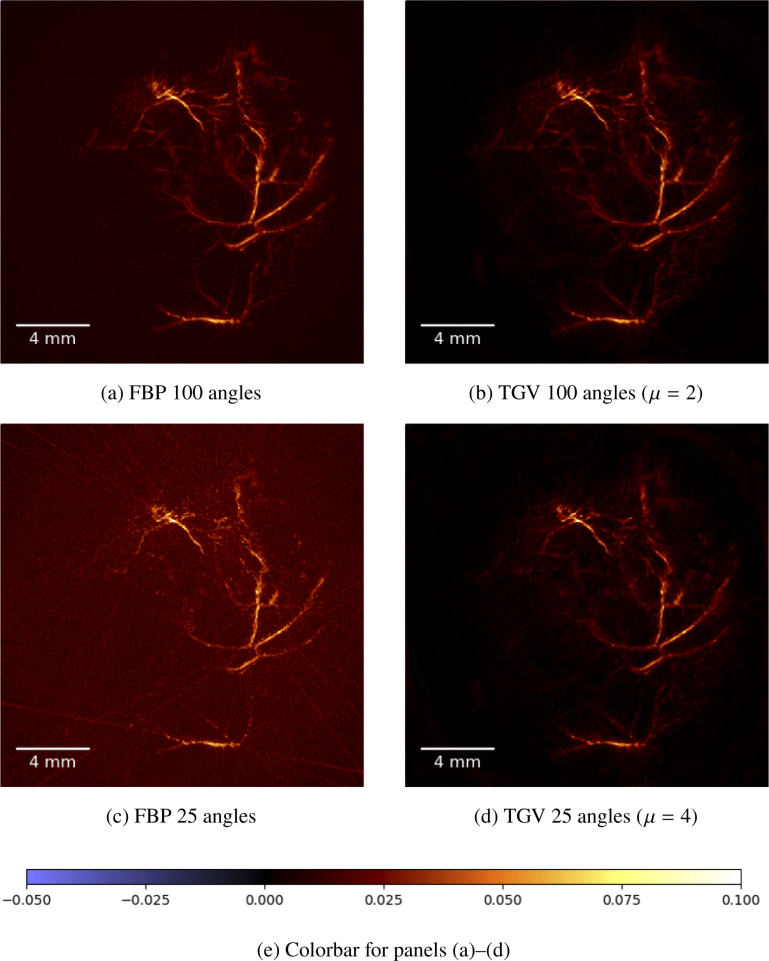
(a)–(d) Maximum amplitude projections along the z-axis of the reconstruction of the in vivo measurements of the hind leg of a mouse. The reconstruction method is indicated below each image, together with the choice of the regularization parameter μ. All images share the same colormap, see (e).

We present results for an in-vivo data set that was obtained by imaging the hind leg of a 14 week old female CD1 mouse. Details about the animal experiment that provided the data set are stated in [11]. The computation is performed on a reconstruction space P of size 571×571×151. The results of the reconstruction of the in vivo measurements are depicted in Figs. 8

and 9

**Fig. 9.**
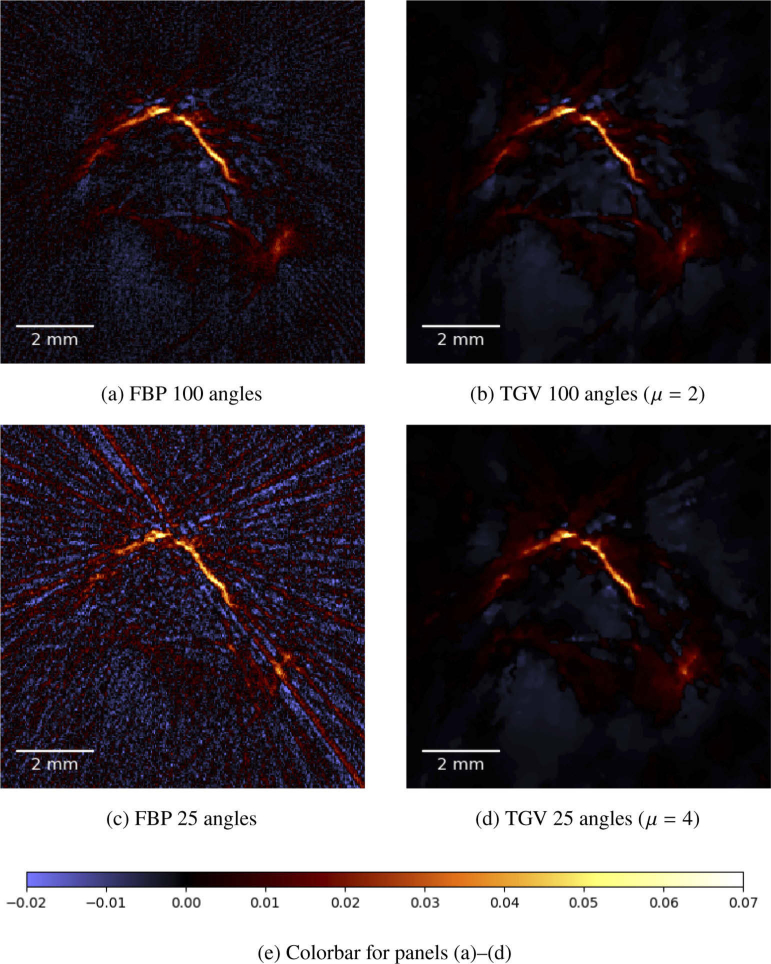
(a)–(d) Close up of a single slice of the reconstruction of the in vivo measurements of the hind leg of a mouse. The reconstruction method is indicated below each image, together with the choice of the regularization parameter μ. All images share the same colormap, see (e). The detail visible in this images is visible in the upper center of the images in Fig. 8.

.

In Fig. 8 we show the maximum amplitude projections along the z-axis for 100 and 25 angles, respectively.

The vasculature structure is clearly visible in the FBP and TGV MAP images when 100 angles are used for the reconstruction (Fig. 8(a) and Fig. 8(b)). As expected, a reduction of the background noise level can be identified using full data with TGV reconstruction compared to FBP. Looking at the results obtained with reduced data (25 angles), the FBP result is strongly affected by stripe artifacts while the TGV method still performs good showing only a minor difference compared to the full-data results. Looking at a certain slice image of the reconstruction, the difference in terms of the noise level and the degree of stripe artifacts between FBP and TGV is even more pronounced (Fig. 9).

## 4. Conclusions and outlook

In this paper we have introduced a reconstruction method for photoacoustic tomography images based on a simple back propagation method and the reconstruction of the initial pressure distribution from the resulting projection data by the inversion of the Radon transform using Tikhonov regularization with TGV as regularization term. The results in Section 3.2 show the benefits of the proposed method. The new method reduces noise significantly and eliminates also some other artifacts (e.g. stripe artifacts) which arise when using FBP for the inversion of the Radon transform. In addition, the new method allows for a reduction of the number of angles in the measurement process: The experiments show that even when only 25% of the angular data is used, almost all features are still recovered while noise is still reduced significantly. If a FBP algorithm is used instead of the proposed method, the reduction of the angles introduces higher noise levels and stripe artifacts, which render the results useless. However, also in case of TGV reconstruction, small features in single slices can suffer from reduced brightness or even be lost when the number of angles is reduced. Hence, a drastic reduction of the angles is reasonable only in applications where small details are of minor interest.

In future works it would be of interest to study the influence of randomly chosen non-uniform distributions of the angles on the proposed method instead of uniform distributions. Indeed, as mentioned before, randomized sampling strategies are successfully considered in the field of compressed sensing, for example in [32] in the context of PAT. Another possible extension of the proposed reconstruction method would be a regularization of the full inversion process, i.e. including the backwards wave propagation. This could help to eliminate further artifacts and increase the quality of the reconstruction even for a reduced number of angles. However, there are several challenges concerning the development of such an algorithm. In particular, very fast implementations of a forward propagation algorithm and its adjoint are needed due to the iterative nature of the considered algorithm for the regularized inversion. Hence, this is open for future work.

## A. Appendix

### A.1. Discretization of the differential operators

In Section 2.2, the discrete differential operators ∇, E and div are introduced and mentioned that these operators can be implemented using a standard finite difference approach. We will give the details of this implementation here.

Let the spaces P, V and W be given by Eq. (13). Recall that we write an element p∈P in the form ${\left(p^{x,y,z}\right)}_{x=0,y=0,z=0}^{N_{x}-1,N_{x}-1,N_{z}-1}$. We define the forward differential operator $\delta_{x}^{+}\;:\;P\to P$ by

$${\left(\delta_{x}^{+}p\right)}^{x,y,z}:=\left\{ \begin{array}{ll} p^{x+1,y,z}-p^{x,y,z}, & \text{if }x\in \{0,\ldots ,N_{x}-2\},\\ 0, & \text{otherwise}, \end{array}\right.$$

and the backward differential operator $\delta_{x}^{-}\;:\;P\to P$ by

$${\left(\delta_{x}^{-}p\right)}^{x,y,z}:=\left\{ \begin{array}{ll} p^{x,y,z}-p^{x-1,y,z}, & \text{if }x\in \{1,\ldots ,N_{x}-1\},\\ 0, & \text{otherwise}. \end{array}\right.$$

The two forward differential operators $\delta_{y}^{+}$, $\delta_{z}^{+}\;:\;P\to P$ and the two backward differential operators $\delta_{y}^{-}$, $\delta_{z}^{-}\;:\;P\to P$ are defined analogously. Using these operators we can define the discrete gradient ∇:P→V and the discrete symmetrized Jacobian E:V→W by

(26) $$\nabla p:=\left(\delta_{x}^{+}p,\delta_{y}^{+}p,\delta_{z}^{+}p\right),$$

(27) $$Eq:=\left(\delta_{x}^{-}q^{1},\delta_{y}^{-}q^{2},\delta_{z}^{-}q^{3},\frac{\delta_{x}^{-}q^{2}+\delta_{y}^{-}q^{1}}{2},\frac{\delta_{x}^{-}q^{3}+\delta_{z}^{-}q^{1}}{2},\frac{\delta_{y}^{-}q^{3}+\delta_{z}^{-}q^{2}}{2}\right).$$

The operators div:V→P and div:W→V have to satisfy $div=-\nabla^{*}$ and $div=-E^{*}$, respectively. For this purpose we introduce the forward differential operator $\delta_{x}^{+,*}\;:\;P\to P$ by

$${\left(\delta_{x}^{+,*}p\right)}^{x,y,z}:=\left\{ \begin{array}{ll} p^{x+1,y,z}-p^{x,y,z} & \text{if }x\in \{1,\ldots ,N_{x}-2\},\\ p^{1,y,z}, & \text{if }x=0,\\ -p^{N_{x}-1,y,z}, & \text{if }x=N_{x}-1, \end{array}\right.$$

and the backward differential operator $\delta_{x}^{-,*}\;:\;P\to P$ by

$${\left(\delta_{x}^{-,*}p\right)}^{x,y,z}:=\left\{ \begin{array}{ll} p^{x,y,z}-p^{x-1,y,z}, & \text{if }x\in \{1,\ldots ,N_{x}-2\},\\ p^{0,y,z}, & \text{if }x=0,\\ -p^{N_{x}-2,y,z}, & \text{if }x=N_{x}-1. \end{array}\right.$$

With these definitions it holds ${\left(\delta_{x}^{+}\right)}^{*}=-\delta_{x}^{-,*}$ and ${\left(\delta_{x}^{-}\right)}^{*}=-\delta_{x}^{+,*}$. Again, we define the forward differential operators $\delta_{y}^{+,*}$, $\delta_{z}^{+,*}\;:\;P\to P$ and the two backward differential operators $\delta_{y}^{-,*}$, $\delta_{z}^{-,*}\;:\;P\to P$ analogously. Then, the divergence operators defined by

$$\begin{array}{l} \mathrm{div}v=\delta_{x}^{-,*}v^{1}+\delta_{y}^{-,*}v^{2}+\delta_{z}^{-,*}v^{3},\\ \mathrm{div}w=\left(\delta_{x}^{+,*}w^{1}+\delta_{y}^{+,*}w^{4}+\delta_{z}^{+,*}w^{5},\;\delta_{x}^{+,*}w^{4}+\delta_{y}^{+,*}w^{2}+\delta_{z}^{+,*}w^{6},\;\delta_{x}^{+,*}w^{5}+\delta_{y}^{+,*}w^{6}+\delta_{z}^{+,*}w^{3}\right), \end{array}$$

satisfy the desired adjointness properties.

### A.2. Radon inversion algorithm using TV instead of TGV

In Section 2.2, TGV is used as a regularizer to solve the discrete version of the minimization problem (7). Here, we will give the details about the necessary changes if TV is used instead of TGV. The minimization problem we consider is

(28) $$\underset{p\in P}{\text{min}}\;\frac{\mu }{2}{\|Rp-f\|}^{2}+\alpha {\left\|\nabla p\right\|}_{\ell^{1}}.$$

for α>0. We use ${\left\|\nabla p\right\|}_{\ell^{1}}$ as a discretization of TV, which can be motivated as in the case of TGV. By defining the spaces $H_{1}=P$ and $H_{2}=S\times V$, as well as the operators $F\;:\;H_{1}\to {\mathbb{R}}^{\infty }$, $G\;:\;H_{2}\to {\mathbb{R}}^{\infty }$ and $A\;:\;H_{1}\to H_{2}$ by

$$F(p)=0,\;G(g,v)=\frac{\mu }{2}{\left\|g-f\right\|}^{2}+\alpha {\left\|v\right\|}_{\ell^{1}},\;A=(Rp,\nabla p),$$

we see that we have again a problem of the form (11). Similarly as in Section 2.2, one can see that

(29) $$A^{*}(g,v)=R^{*}g-\mathrm{div}v,$$

(30) $${\text{prox}}_{\tau }^{F}(p)=p,\;{\text{prox}}_{\sigma }^{G^{*}}(g,v)=\left(\frac{\mu }{\mu +\sigma }(g-\sigma f),\;{\text{proj}}_{\left\{\bar{v}\in V\;:\;{\left\|\bar{v}\right\|}_{\ell^{\infty }}\leq \alpha \right\}}(v)\right),$$

and by assuming ‖R‖≤1, one can show that ${\left\|A\right\|}^{2}<9$.

This allows us to apply Algorithm 1, which can be rewritten in the form of Algorithm 3. It yields the approximate solution $p^{N}$.

**Algorithm 3:** Primal Dual Algorithm for the solution of (28)

Estimate ∥**R**∥ using the power iteration on **R*****R** and scale **R**, **R*** and *f* by 1/∥**R**∥ if necessary. **Require:** Parameters *σ*, *τ*>0, s.t. $\sigma \tau <\frac{1}{9}$ **Initialize:** *p*^0^ = 0, ${\bar{p}}^{0}=0\;\text{in}\;P$ , *v*^0^ = 0 in *V*, *g*^0^ = 0 in *S* **for** *n* = 0, 1, …, *N* – 1 **do** Dual Update: $$\begin{array}{l} g^{n+1}=\frac{\mu }{\sigma +\mu }(g^{n}+\sigma (R\;{\bar{p}}^{n}-f))\\ v^{n+1}={\text{proj}}_{\left\{\bar{v}\in V:\;{\left\|\bar{v}\right\|}_{\ell^{\infty }}\leq \alpha \right\}}(v^{n}+\sigma \nabla {\bar{p}}^{n}) \end{array}$$ Primal Update: $$p^{n+1}=p^{n}-\tau (R^{*}g^{n+1}-\text{div}\;v^{n+1})$$ Extragradient Update: $${\bar{p}}^{n+1}=2p^{n+1}-p^{n}$$ **end for** **return** *p^N^*
